# Acute changes in forearm vascular compliance during transient sympatho‐excitation

**DOI:** 10.14814/phy2.15256

**Published:** 2022-04-19

**Authors:** T. Dylan Olver, Mark B. Badrov, Matti D. Allen, Nicole S. Coverdale, J. Kevin Shoemaker

**Affiliations:** ^1^ 7235 Biomedical Sciences Western College of Veterinary Medicine University of Saskatchewan Saskatoon Saskatchewan Canada; ^2^ Division of Cardiology Department of Medicine University Health Network and Sinai Health University of Toronto Toronto Ontario Canada; ^3^ 4257 Department of Physical Medicine and Rehabilitation School of Medicine Queen's University Kingston Ontario Canada; ^4^ School of Kinesiology and Health Studies Queen’s University Kingston Ontario Canada; ^5^ Neurovascular Research Laboratory School of Kinesiology The University of Western Ontario London Ontario Canada

**Keywords:** peripheral blood flow, resistance, sympatho‐excitation, vascular compliance

## Abstract

The study of vascular regulation often omits important information about the elastic properties of arteries under conditions of pulsatile flow. The purpose of this study was to examine the relationship between muscle sympathetic nerve activity (MSNA), vascular bed compliance, and peripheral blood flow responses in humans. We hypothesized that increases in MSNA would correlate with reductions in vascular compliance, and that changes in compliance would correspond with changes in peripheral blood flow during sympatho‐excitation. MSNA (microneurography), blood pressure (Finopres), and brachial artery blood flow (Doppler ultrasound), were monitored in six healthy males at baseline and during the last 15 s of voluntary end‐inspiratory, expiratory apneas and 5 min of static handgrip exercise (SHG; 20% maximum voluntary contraction) and 3 min of post‐exercise circulatory occlusion (SHG + PECO; measured in the non‐exercising arm). A lumped Windkessel model was employed to examine vascular bed compliance. During apnea, indices of MSNA were inversely related with vascular compliance, and reductions in compliance correlated with decreased brachial blood flow rate. During SHG, despite increased MSNA, compliance also increased, but was unrelated to increases in blood flow. Neither during SHG nor PECO did indices of MSNA correlate with forearm vascular compliance nor did vascular compliance correlate with brachial flow. However, during PECO, a linear combination of blood pressure and total MSNA was correlated with vascular compliance. These data indicate the elastic components of the forearm vasculature are regulated by adrenergic and myogenic mechanisms during sympatho‐excitation, but in a reflex‐dependent manner.

## Graphical abstract

During apnea, increases in sympathetic nerve activity were related to reductions in vascular compliance, with such reductions impeding flow rate. During post‐exercise circulatory occlusion, both sympathetic factors and blood pressure appeared to modulate vascular compliance (data not shown in graphical abstract). Vascular compliance appears to be regulated by adrenergic and myogenic mechanisms during sympatho‐excitation, but in a reflex‐dependent manner.

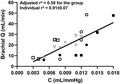

## 1 INTRODUCTION

Sympathetic neurovascular control represents a major feature by which the autonomic nervous system regulates homeostasis through the reflexive regulation of blood pressure and blood flow. This process, in turn, involves the transduction of efferent post‐ganglionic sympathetic nerve activity (SNA) into a subsequent vascular response at the end‐organ (i.e., neurovascular transduction). Sympathetic outflow directed to the skeletal muscle vasculature (muscle SNA; MSNA) occurs in “bursts” of activity, reflecting periods of efferent action potential synchronization. At baseline, these occur at an incidence of ~20–30 bursts·100 heart beats^−1^ in young healthy individuals (Fairfax, Holwerda, et al., 2013; Fairfax, Padilla, Vianna, Holwerda, et al., 2013; Steinback et al., 2010; Vianna et al., 2012), with MSNA activity increasing ~two‐ or three‐fold during states of autonomic arousal, such as that during systemic hypoxia or hypercapnia (Badrov et al., 2015; Morgan et al., 1995; Steinback et al., 2010) and fatiguing exercise (Zamir et al., 2017). Likewise, during autonomic arousal the rate or probability of neural firing or the amplitude of sympathetic bursts through axonal recruitment increases (Badrov et al., 2015; Steinback et al., 2010). Under resting conditions, spontaneously occurring bursts of MSNA do not necessarily mediate constriction of conduit arteries (Fairfax, Padilla, Vianna, Holwerda, et al., 2013), but do mediate constriction in the downstream vascular bed, as indicated by increased vascular resistance (Davy et al., 1998; Fairfax, Holwerda, et al., 2013; Fairfax, Padilla, Vianna, Davis, et al., 2013; Fairfax, Padilla, Vianna, Holwerda, et al., 2013; Grassi et al., 1995; Lundvall & Edfeldt, 1994; Ray & Monahan, 2002; Steinback et al., 2010; Tan, Tzeng, et al., 2013; Vianna et al., 2012; Vissing et al., 1989; Wahlestedt & Hakanson, 1990).

The in vivo study of the neurogenic control of the skeletal muscle vasculature focuses primarily on changes in vascular bed resistance (Davy et al., 1998; Fairfax, Holwerda, et al., 2013; Fairfax, Padilla, Vianna, Holwerda, et al., 2013; Lundvall & Edfeldt, 1994; Ray & Monahan, 2002; Vianna et al., 2012; Vissing et al., 1989). From a conceptual standpoint, neural‐mediated changes in vessel diameter produce changes in the resistance to flow, and therefore, flow rate and fluid conductance. However, this model represents an oversimplification of neurovascular transduction, as it assumes steady‐state conditions of flow. Rather, the cardiovascular system is pulsatile, and therefore, when examining the pulsatile characteristics of flow, this model fails to adequately describe the mechanical properties of blood vessels. Consider that in an exclusively resistive system, blood pressure and blood flow waveforms would appear identical. However, this does not occur under normal physiological circumstances, highlighting a limitation to examining the neurogenic control of vasomotor tone using a resistive model alone (Zamir et al., 2007, 2009). Indeed, central artery stiffening or stiffening of conduit arteries is associated with elevated resting MSNA and has likewise been observed during periods of elevated SNA (Boutouyrie et al., 1994; Grassi et al., 1995; Holwerda et al., 2019). Therefore, changes in vascular compliance should be considered when quantifying neurovascular transduction.

Vascular compliance, which addresses vessel wall elastic properties under conditions of pulsatile flow, is affected by vascular smooth muscle contraction (Grassi et al., 1995; Zamir et al., 2007), and may offer additional insight into the neurogenic control of the skeletal muscle vasculature. Vascular compliance appears to be regulated dynamically (Grassi et al., 1995; Moir et al., 2020), and similar to how a reduction in resistance vessel diameter increases vascular resistance, a reduction in vascular bed compliance increases vascular bed impedance (Zamir et al., 2007). In both cases, the net result would be a reduction in flow rate through the vascular bed at a given blood pressure. Whereas the relationship between sympatho‐excitatory reflexes, vascular resistance, and peripheral blood flow has been studied extensively (Joyner & Casey, 2015; Shoemaker et al., 2018), the relationship between sympatho‐excitatory reflexes, vascular bed compliance, and peripheral blood flow has received less attention.

Therefore, the major aim of this study was to examine the effect of transient sympatho‐excitation on acute changes in forearm vascular bed compliance. To elicit large, reliable increases in MSNA, we utilized maximal voluntary end‐inspiratory (EIA) and end‐expiratory apneas (EEA), as well as static handgrip (SHG) and post‐exercise circulatory occlusion (PECO; metaboreflex stimulus). We hypothesized that increased MSNA would correlate with reductions in vascular bed compliance, and that changes in vascular bed compliance would be related to changes in peripheral blood flow during sympatho‐excitation.

## 2 METHODS

### 2.1 Participants

This was a pilot study aimed at determining the effect of acute sympatho‐excitation on peripheral vascular compliance using methods previously established in our laboratory (Zamir et al., 2007, 2009). As such, a small homogenous group was studied. It consisted of six healthy, Caucasian male subjects (age = 27 ± 3 years, height = 180 ± 6 cm, mass = 86 ± 5 kg). Subjects were recreationally active (non‐systematically trained), non‐smoking, and otherwise healthy (i.e., free of known cardiometabolic disease, as assessed by a standardized health questionnaire). All experimental procedures and potential risks were explained fully to the subjects prior to participation and all subjects provided written, informed consent. This study was approved by the Health Sciences Research Ethics Board at Western University (REB:102782/17810).

### 2.2 Experimental protocol

Participants arrived at the laboratory having fasted (12 h) and abstained from alcohol (24 h), caffeine (12 h), and exercise (24 h) prior to data collection. Data were collected during a 3 min baseline period (spontaneous breathing) prior to, as well as between, the following sympatho‐excitatory maneuvers: A maximal voluntary EIA, EEA, and SHG + PECO protocol. Subjects were instructed to continue the breath holds for as long as possible to elicit the greatest increase in MSNA. The average duration of the EIA was 87 ± 38 s, and the average duration of the EEA was 31 ± 11 s. The last 15 s of each breath hold was used for analyses. The order of breath holds was rotated systematically and preceded the SHG + PECO protocol. Prior to the SHG + PECO protocol, participants completed two maximum voluntary contractions with their non‐dominant hand (left hand for all participants). Following a 3 min baseline period, participants performed a 5‐min SHG performed at 20% of the greatest maximum voluntary contraction recorded (Zamir et al., 2017). The SHG was followed immediately by a 3 min period of PECO (Zamir et al., 2017). Forearm circulatory occlusion was initiated 5 s prior to the end of SHG and achieved by rapidly inflating a pneumatic cuff (Hokanson SC12D, D.E. Hokanson, Inc.) on the upper exercised arm to a suprasystolic blood pressure (~200 mmHg). The last 15 s of SHG prior to PECO, and the last 15 s of PECO were used for analyses.

### 2.3 Experimental measures

Muscle sympathetic nerve activity was recorded from the right peroneal nerve using microneurography (Hagbrath & Vallbo, 1968). Briefly, a tungsten microelectrode (~35 mm long with a 1–5 µm diameter uninsulated tip) was inserted transcutaneously into the peroneal nerve and a reference electrode was positioned subcutaneously 1–3 cm from the recording site. Verification that the signal reflected MSNA was determined by the presence of a characteristic pulse‐synchronous burst pattern and an increase in activity to voluntary apnea, as well as the absence of skin paresthesia and a lack of response during arousal to loud noise (Delius et al., 1972). The obtained MSNA signal was pre‐amplified (1000×) and further amplified (75×) by a variable gain amplifier (662C‐3, Department of Bioengineering, University of Iowa). The resultant raw MSNA signal was band‐pass filtered (bandwidth of 700–2000 Hz), rectified and integrated (leaky integrator; 0.1 s time constant). The MSNA signals were sampled at 10000 Hz and stored for further analysis (LabChart 7 and PowerLab Data Acquisition System, ADInstruments). Muscle sympathetic nerve activity was analyzed from the integrated neurogram as described previously (Badrov et al., 2015) and quantified using burst frequency (the number of bursts per minute) and burst incidence (the number of bursts per 100 heart beats [hb]), as well as burst amplitude (normalized to the largest recorded burst at baseline, which was given a value of 100) and total MSNA (the product of burst frequency and burst amplitude).

Heart rate (HR) was measured using a three‐lead electrocardiogram. Resting blood pressure was obtained in the right brachial artery by manual sphygmomanometry. Thereafter, beat by beat blood pressure during the experimental protocol was monitored from the right middle finger by photoplethysmography (Finometer; Finapres Medical Systems), and brachial artery blood pressure was determined via waveform reconstruction. Finometer blood pressure values were corrected to the baseline manual sphygmomanometric values. Additionally, cardiac output (Q) was determined by the Finometer Modelflow algorithm that integrates sex, age, height, and weight. Stroke volume (SV) was calculated as the quotient of Q and HR. Total peripheral resistance (TPR) was calculated as the quotient of mean arterial pressure and Q. Concurrently, right brachial diameter and blood flow velocity (the inactive arm during SHG + PECO) were assessed using Doppler ultrasound in duplex imaging mode (4 MHz Doppler ultrasound; 10 MHz ultrasound imaging; GE Vivid 7; Mississauga, ON, Canada). Brachial blood flow was calculated as the product of the measured velocity and cross‐sectional area of the artery, as determined from the measured diameter (assessed with calipers at end‐diastole at three regions along the brachial artery and averaged; GE Echopac Software). Heart rate, Q, SV, mean arterial pressure (MAP), and blood flow velocities were collected and analyzed with a data analysis software program (LabChart 7, PowerLab, ADInstruments).

Forearm vascular mechanics (i.e., resistance and compliance) were analyzed using a modified lumped Windkessel model as described previously in detail (Zamir et al., 2007, 2009). Briefly, the dynamics of pulsatile flow through a vascular network are influenced by mechanical properties, chiefly resistance, and compliance. The resistance value is obtained by measuring MAP and mean flow at the entry point of the vascular bed. The compliance values are calculated from the oscillatory pressure and flow waveforms at the entry point of the vascular bed. It has been demonstrated that resistance and compliance contribute to the regulation of flow and pressure within the vascular bed and operate independently (Zamir et al., 2007).

### 2.4 Statistical analyses

Analyses were completed using Sigma Stat software (version 14) and graphs were prepared using Graphpad Prism software (version 9.2). Paired, two‐tailed *t*‐tests were used to compare hemodynamic and MSNA variables obtained at baseline versus the end of either EIA or EEA. A one‐way repeated measures ANOVA was used to compare hemodynamic and MSNA variables at baseline, during SHG and PECO, with planned comparisons for SHG and PECO versus baseline analyzed using a Dunnett's test. Cohen's d effect sizes were calculated (Cohen, 1988; Sawilowsky, 2009) to determine the magnitude and direction of change in MSNA, brachial blood flow, vascular resistance, and compliance during sympatho‐excitation. Pearson's correlation coefficients were used to examine the relationship between indices of MSNA and forearm vascular resistance and compliance during apnea (data for EIA and EEA were grouped as they had a similar effect on indices of MSNA, vascular resistance, compliance and brachial blood flow) as well as SHG and PECO. Based on the results of the linear regressions, a multiple linear regression analysis was used to examine whether a linear combination of total MSNA and MAP was correlated with vascular compliance during PECO. Pearson's correlation coefficients were used to examine the relationship between vascular compliance and brachial blood flow. Based on the results of the linear regressions, coefficients of determination were calculated for the pooled data as well as individual responses to examine the relationship between forearm vascular resistance and forearm blood flow as well as forearm vascular compliance and blood flow during apnea. Significance was set at *p* ≤ 0.05 with trends interpreted at *p* = 0.06–0.07. Where possible, individual data are plotted with mean data (all individual data collected are presented). Otherwise, data are presented as mean ± SD.

## 3 RESULTS

### 3.1 Systemic hemodynamics

During EIA, Q, and SV decreased, whereas SBP, DBP, and TPR increased (all *p* ≤ 0.01; Table 1). Conversely, HR did not change (*p* = 0.38; Table 1). During EEA, Q, HR, SBP, and TPR remained unchanged (*p* ≥ 0.10), increases in DBP approached significance (*p* = 0.07) and SV decreased (*p* = 0.05; Table 1). From baseline to the end of SHG, Q, HR, and DBP increased (*p* ≤ 0.04), but SV, SBP, and TPR did not change significantly (*p* ≥ 0.11; Table 1). At the end of PECO, Q, HR, and DBP remained elevated and SBP as well as TPR were increased compared to baseline values (*p* ≤ 0.04; Table 1).

**TABLE 1:** Systemic hemodynamic variables

	Q (L/min)	HR (bpm)	SV (ml/min)	SBP (mmHg)	DBP (mmHg)	TPR (mmHg/ml/min)
EIA						
Base	7.0 ± 2.9	65 ± 4	113 ± 16	127 ± 8	77 ± 9	13 ± 3
End	5.7 ± 2.5*	62 ± 8	95 ± 23*	133 ± 8*	85 ± 9*	18 ± 4*
EEA						
Base	7.4 ± 1.4	64 ± 5	115 ± 18	126 ± 7	76 ± 8	13 ± 3
End	7.0 ± 1.5	70 ± 8	100 ± 23*	129 ± 9	82 ± 11	15 ± 4
SHG + PECO						
Base	6.0 ± 0.8	62 ± 4	98 ± 16	129 ± 12	81 ± 11	16.6 ± 3.8
SHG	7.7 ± 1.5*	80 ± 10*	96 ± 19	143 ± 11	92 ± 11*	15.2 ± 3.7
PECO	7.0 ± 1.29*	68 ± 2*	102 ± 19	137 ± 11*	86 ± 10*	15.7 ± 3.9*

Abbreviations: DBP, diastolic blood pressure; EEA, end‐expiratory apnea; EIA, end‐inspiratory apnea; HR, heart rate; PECO, post‐exercise circulatory occlusion Base, baseline; Q, cardiac output; SBP, systolic blood pressure; SHG, static handgrip; SV, stroke volume; TPR, total peripheral resistance.

* Significantly different from baseline (*p* < 0.05).

### 3.2 Muscle sympathetic nerve activity and forearm vascular characteristics

**FIGURE 1:**
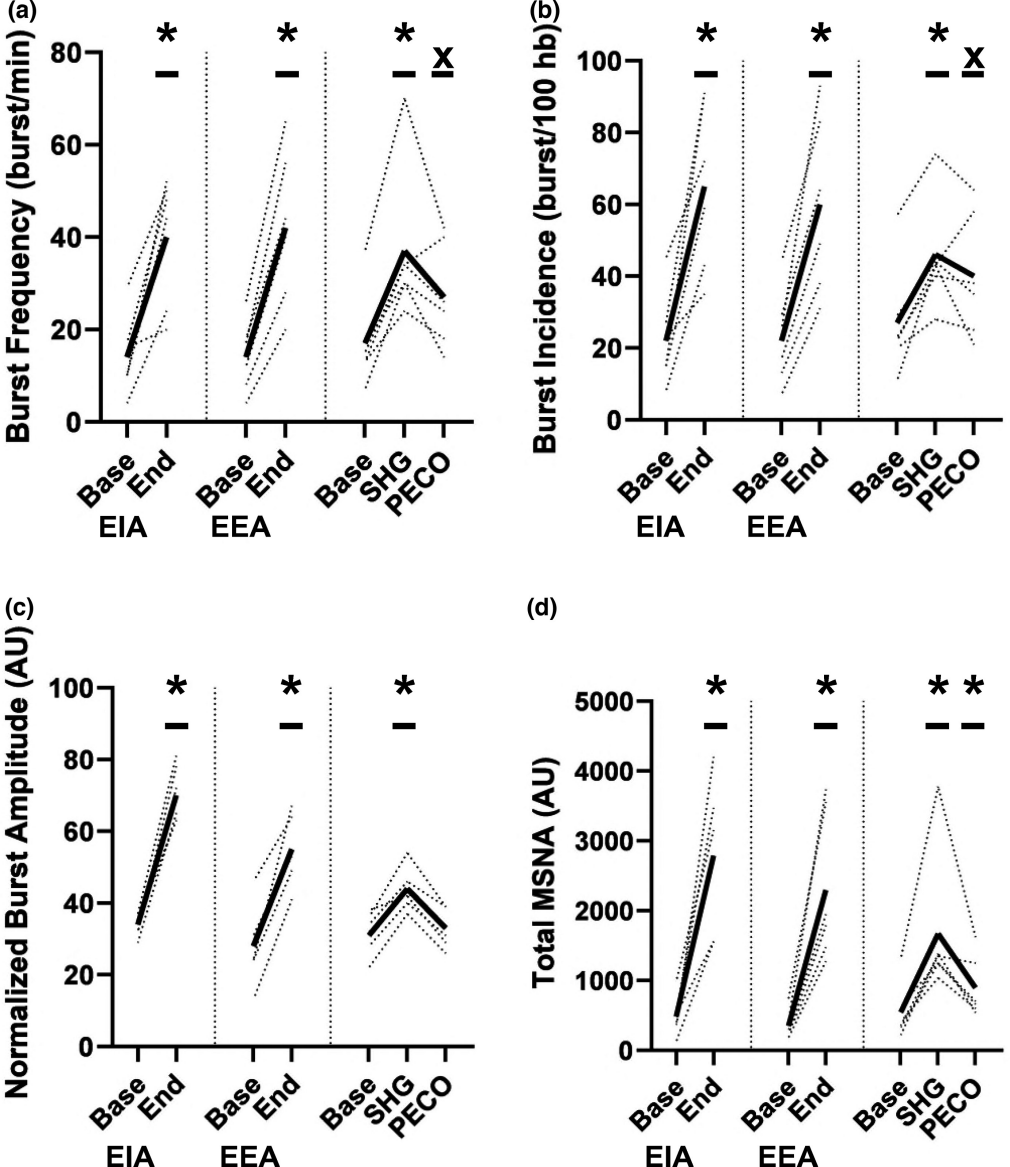
Indices of muscle sympathetic nerve activity (MSNA); (a) burst frequency, (b) burst incidence, (c) normalized burst amplitude and (d) total MSNA, at baseline and during end inspiratory apnea (EIA), end expiratory apnea (EEA), end static handgrip exercise (SHG) and post‐exercise circulatory occlusion (PECO). EIA and EEA data analyzed using a paired, two‐tail *t*‐test, and SHG + PECO data analyzed using a one‐way repeated measures ANOVA. *, Significantly different from baseline (*p* < 0.05), X, *p* = 0.05–0.07 versus baseline. Dotted lines are individual responses (*n* = 6) and dark, bold lines are mean data

During EIA, EEA, and SHG all indices of MSNA increased significantly (all *p* ≤ 0.01; Figure 1a–d). Elevations in burst frequency and incidence approached significance during PECO (*p* ≤ 0.07), normalized burst amplitude returned to pre‐SHG values (*p* = 0.81), and total MSNA remained elevated during PECO (*p* = 0.04; Figure 1a–d). The magnitude and direction of changes in indices of MSNA during apnea and SHG as well as PECO are presented in Table 2. Of note, the effect of EIA and EEA on indices of MSNA were directionally the same and similar in magnitude.

**TABLE 2:** Effect size table

	EIA	EEA	SHG	PECO
Burst Frequency (burst/min)	1.5^a^	1.4^a^	1.2^a^	0.9^d^
Burst Incidence (burst/100hb)	1.5^a^	1.4^a^	1.0^a^	0.8^d^
Normalized Burst Amp. (%)	1.8^a^	1.6^a^	1.4^a^	0.3^d^
Total MSNA (AU)	1.6^a^	1.6^a^	1.2^b^	0.8^d^
Brachial blood flow (ml/min)	−1.6^a^	−1.3^a^	1.0^c^	−0.3^c^
Forearm vascular R (mmHg/ml/min)	1.6^a^	1.4^b^	−0.6^d^	0.7^d^
Forearm vascular C (ml/mmHg)	−1.2^a^	−1.0^a^	0.7^a^	−0.1^d^

Note

Effect size scale: small = 0.2–0.49, medium = 0.5–0.79, large ≥ 0.8. Power (β) is reported.

Abbreviations: C, compliance; EEA, end‐expiratory apnea; EIA, end‐inspiratory apnea; PECO, post‐exercise circulatory occlusion; R, resistance; SHG, static handgrip.

^a^ Signifies *β* > 0.9.

^b^ Signifies *β* = 0.8–0.89.

^c^ Signifies *β* = 0.7–0.79.

^d^ Signifies *β* < 0.7.

**FIGURE 2:**
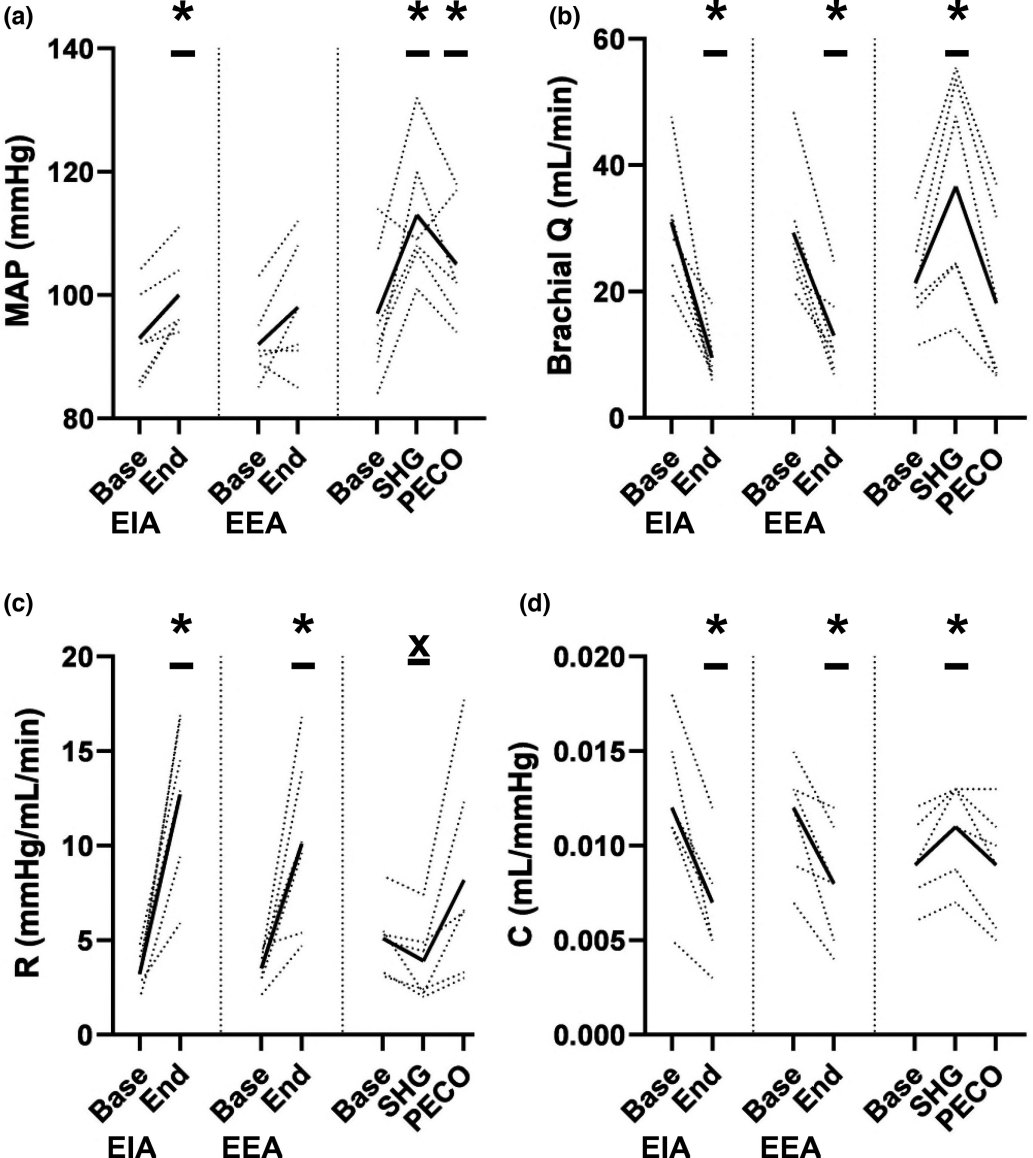
Hemodynamics; (a) mean arterial pressure (MAP), (b) brachial artery blood flow (Q), (c) forearm vascular resistance (R) and (d) forearm vascular compliance (C) at baseline and during end inspiratory apnea (EIA), end expiratory apnea (EEA), end static handgrip exercise (SHG) and post‐exercise circulatory occlusion (PECO). EIA and EEA data analyzed using a paired, two‐tail *t*‐test, and SHG+PECO data analyzed using a one‐way repeated measures ANOVA. *, Significantly different from baseline (*p* < 0.05), X, *p* = 0.06 versus baseline. Dotted lines are individual responses (*n* = 6) and dark, bold lines are mean data

During EIA, MAP increased significantly (*p* < 0.01), but during EEA it did not (*p* = 0.13; Figure 2a). During both EIA and EEA brachial blood flow decreased, forearm vascular resistance increased and vascular compliance decreased (*p* ≤ 0.02; Figure 2b–d). Relative to baseline, MAP, brachial blood flow, and forearm vascular compliance were increased at the end of SHG (*p* ≤ 0.04), while reductions in forearm vascular resistance only approached significance (*p* = 0.06; Figure 2a–d). Although MAP remained elevated at the end of PECO (*p* < 0.01), brachial blood flow, forearm vascular resistance and compliance returned to pre‐SHG values (*p* ≥ 0.20; Figure 2a–d). The magnitude and direction of changes in brachial blood flow, vascular resistance and compliance during apnea and SHG as well as PECO are presented in Table 2. Of note, the effect of EIA and EEA on brachial blood flow, vascular resistance and compliance were directionally the same and similar in magnitude.

### 3.3 Neurovascular and hemodynamic relationships

Pearson's correlation coefficients were calculated to examine the relationship between MSNA and forearm vascular mechanics. The data reveal that during apnea, all indices of MSNA were positively correlated with forearm vascular resistance and negatively correlated with compliance (*p* < 0.05; Table 3). Overall, the relationships were stronger between indices of MSNA and resistance versus compliance. Neither during SHG nor PECO did indices of MSNA correlate with forearm vascular resistance or compliance (*p* ≥ 0.14; Table 3). Based on previous evidence that vascular mechanics in a non‐exercising limb may be related more to MAP than total MSNA during PECO (Shoemaker et al., 2000), linear regression and multiple linear regression analyses were used post hoc to examine the relationship between MAP and compliance as well as MAP + total MSNA and compliance. The data reveal that MAP alone was not correlated with compliance during PECO (*p* = 0.13); however, a linear combination of MAP (coefficient: −0.00214; *p* < 0.01) and total MSNA (coefficient: 0.00000456; *p* = 0.02) was correlated with compliance during PECO (adjusted r^2^ = 0.51; *p* = 0.02; *β* = 0.87).

**TABLE 3:** Linear regression correlation coefficients for indices of MSNA versus forearm vascular R and C

	Burst frequency (burst/min)			Burst incidence (burst/100 hb)			Normalized burst amp. (%)			Total MSNA (AU)		
	Apnea	SHG	PECO	Apnea	SHG	PECO	Apnea	SHG	PECO	Apnea	SHG	PECO
FA vascular R (mmHg/ml/min)	0.62*	−0.42	−0.11	0.70*	−0.30	−0.13	0.74*	−0.26	−0.02	0.74*	−0.39	−0.15
FA vascular C (ml/mmHg)	−0.49*	0.20	0.15	−0.49*	0.26	0.16	−0.56*	0.22	−0.25	−0.57*	0.17	0.06

Abbreviations: AU, arbitrary units; C, compliance; FA, forearm; hb, heart beats; MSNA, muscle sympathetic nervous activity; PECO, post‐exercise circulatory occlusion; R, resistance; SHG, static handgrip.

* Significantly related (*p* < 0.05).

**FIGURE 3:**
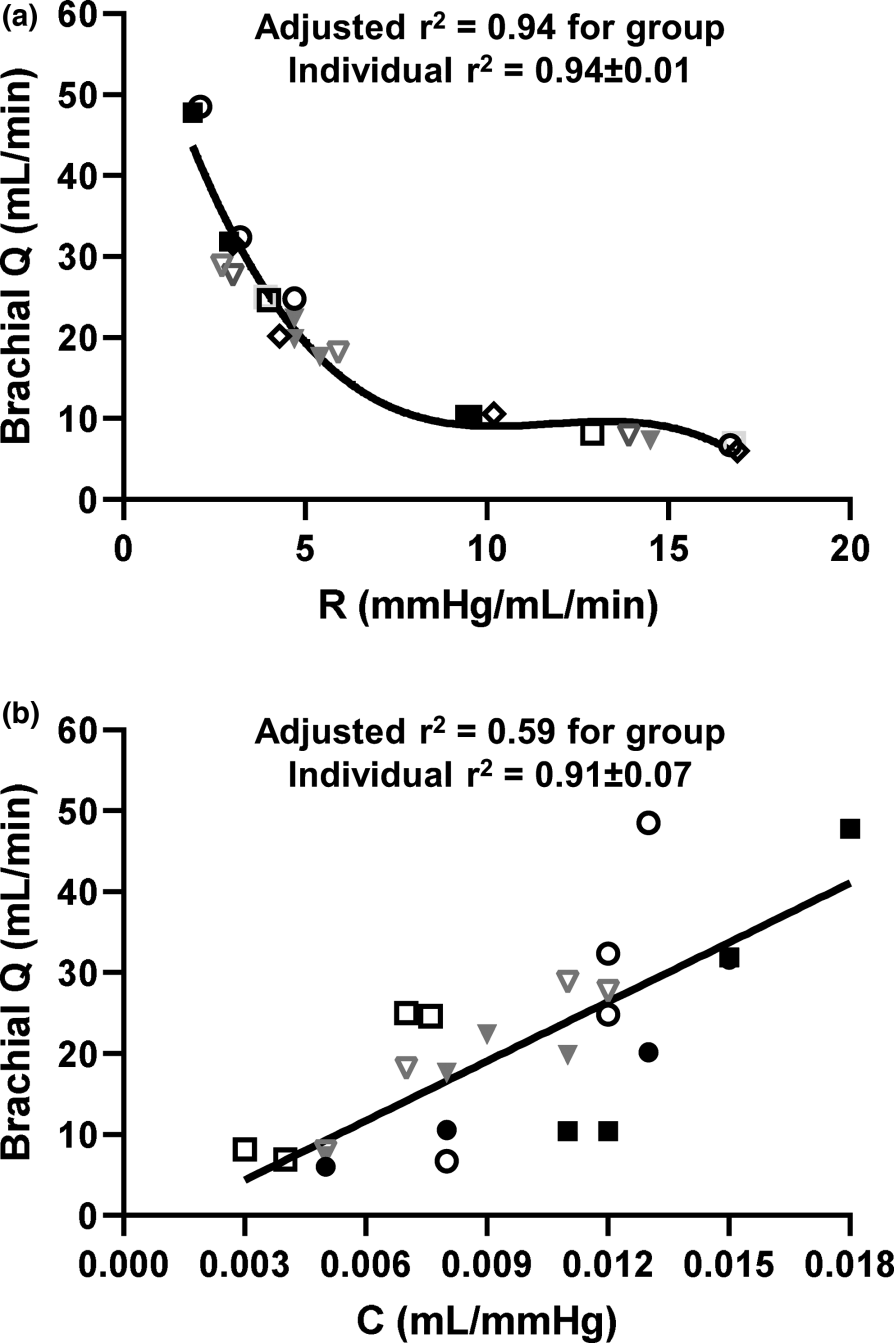
Hemodynamic relationships during end inspiratory and expiratory apnea; (a) brachial artery blood flow (Q) plotted against forearm vascular resistance (R), (b) brachial artery Q plotted against forearm vascular compliance (C) during end inspiratory and end expiratory apnea. The R‐flow curve was modeled with a 3rd order polynomial and the C‐flow curve was modeled with a linear regression. Symbols represent individual responses (*n* = 6)

Pearson's correlation coefficients were calculated to examine the relationship between vascular compliance and brachial blood flow. The data reveal forearm vascular compliance only correlated significantly with brachial blood flow during apnea (*r* = 0.77; *p* < 0.01) but neither SHG nor PECO (*p* ≥ 0.55). To further interrogate forearm blood flow control during apnea, coefficients of determination (i.e., r^2^) were generated for pooled data as well as based on individual resistance‐flow and compliance ‐flow responses. The best non‐linear fit for resistance and flow was a third order polynomial relationship (pooled data; 1st order: *β*0 = 69.0, *β*1 = −15.8; r^2^ = 0.73; *p* < 0.01; 2nd order: *β*2 = 1.4; r^2^ = 0.16; *p* < 0.01; 3rd order: *β* = −0.04; r^2^ = 0.05; *p* < 0.01; sum: adjusted r^2^ = 0.94; *p* < 0.01), with individual r^2^ values ranging between 0.92 and 0.95 (0.94, 0.94, 0.95, 0.95, 0.94, 0.92) (Figure 3a). The correlation between compliance and flow was expressed as a linear relationship (pooled data; adjusted r^2^ = 0.59; *p* < 0.01) with individual r^2^ values ranging between 0.82 and 0.98 (0.90, 0.83, 0.94, 0.98, 0.98, 0.82) (Figure 3b).

## 4 DISCUSSION

The results of this study reveal several novel features of vascular regulation that advance our understanding of the neurogenic control of the skeletal muscle vasculature. First, we demonstrate that the elastic ability of the forearm vascular bed (associated with compliance) decreases during apnea and increases during SHG. We interpret this reflex‐dependent response to reflect aspects of α‐adrenergic (Frances et al., 2011; Grassi et al., 1995) and potentially β‐adrenergic (Eklund & Kaijser, 1976; Eklund et al., 1974) or cholinergic (Sanders et al., 1989; J K Shoemaker, 2012) control of vascular bed compliance. Second, we found that a linear combination of MAP + MSNA correlated with compliance during PECO. Albeit a small sample size for this particular analysis, we interpret this as evidence of the potential of dual α‐adrenergic and myogenic control of elasticity (Frances et al., 2011). Third, we demonstrate that vascular bed compliance and resistance contribute differentially to forearm blood flow during apnea and SHG + PECO. Thus, we speculate that the elastic ability and vascular volume of the forearm vascular bed may represent distinct sites/mechanisms for neural‐mediated blood flow control. Therefore, both vascular bed compliance and resistance respond to post‐ganglionic efferent MSNA and contribute to changes in forearm blood flow observed during sympatho‐excitation, but in a reflex‐dependent manner.

### 4.1 Apnea and forearm vascular compliance

In the present study, EIA and EEA elicited a robust MSNA response resulting in increased forearm vascular resistance and decreased vascular compliance and blood flow rate. Consider that vascular compliance is a measure of the change in blood vessel luminal volume for a given change in blood pressure. This relationship is not linear, and the compliance of an artery is not constant. Rather, under passive conditions, compliance decreases as blood pressure increases and the artery expands (i.e., volume increases) (Klabunde, 2011; Spencer & Denison, 1963). Herein compliance decreased as blood pressure increased, possibly related to myogenic reactivity; however, vascular compliance was not inversely related to vascular volume, as vascular compliance decreased and resistance increased (reflective of decreased volume), simultaneously, during apnea. Further, reductions in vascular compliance, which were comparable among EIA and EEA and associated with reductions in blood flow, were not driven by cardiac Q (increasing vascular volume), as Q decreased during EIA and remained unchanged during EEA. Thus, changes in forearm vascular compliance were likely the result of neural activity and not passive responses to pressure‐ or Q‐induced increases in vascular volume. Similar observations were made by Holwerda et al. (2019), who reported decreases in common carotid artery compliance coefficient coincided with unchanged MAP and increases in MSNA during lower‐body negative pressure in young adults. Albeit, whereas the current results pertain to transitory increases in SNA and the work by Holwerda et al. (2019) apply to more steady‐state conditions, these data indicate that decreases in both conduit artery and vascular network compliance may be modulated by SNA and not volume expansion of the vasculature.

The current data collected during apnea suggest that reductions in vascular cross‐sectional area and the elasticity of the vascular bed operated in concert to impede flow rate. Changes in arterial (Boutouyrie et al., 1994; Grassi et al., 1995) and vascular bed compliance (Zamir et al., 2007) can occur independently from changes in vascular cross‐sectional area (Boutouyrie et al., 1994; Grassi et al., 1995; Zamir et al., 2007). Concerning a potential mechanism, the evidence supporting independent roles for α‐adrenergic activation versus myogenic reactivity on compliance are equivocal. Previously, Grassi et al. (1995) demonstrated α‐adrenergic‐mediated reductions in radial artery compliance (i.e., α‐adrenergic agonists decrease vascular compliance). However, in our earlier report (Frances et al., 2011), norepinephrine‐induced changes in vascular compliance occurred concurrently with changes in blood pressure (Frances et al., 2011) and correction of that pressure stimulus by positioning the arm above the heart resulted in the restoration of compliance, pointing to a strong myogenic component in the regulation of forearm vascular compliance. Based on the present results, it is possible that reductions in vascular bed compliance, which contributed to reductions in forearm blood flow, during transient apnea‐induced sympatho‐excitation were the result of a combination of α‐adrenergic activation and myogenic reactivity mechanisms.

### 4.2 Static hand grip, PECO, and vascular compliance

In the current study, contrary to our hypothesis, SHG increased forearm vascular compliance in the non‐working arm, despite increases in MSNA, blood pressure, and cardiac Q. Fatiguing static exercise is a unique stimulus whereby increases in blood flow to non‐exercising limbs during SHG may be dissociated from MSNA responses and relate to increased blood pressure, increased circulating catecholamines (Gustafson & Kalkhoff, 1982; Sanchez et al., 1980; Sheehan et al., 1983; Shoemaker et al., 2000), and subsequent β‐adrenergic‐induced vasodilation (Eklund & Kaijser, 1976; Eklund et al., 1974), or possibly a neural‐mediated cholinergic‐dependent vasodilation (Sanders et al., 1989; J K Shoemaker, 2012). These various mechanisms also may influence forearm vascular compliance. Another possibility is that forearm vascular shear stress increased secondary to increases in brachial artery flow, and shear‐induced changes in the phosphorylation status of protein kinase B or endothelial nitric oxide synthase may have facilitated the increased vascular compliance (Gielen et al., 2010; Soucy et al., 2006; Wilkinson et al., 2004). Previously, Tan and coworkers (Tan, Tamisier, et al., 2013) examined neurovascular transduction during SHG exercise and proposed SNA‐induced increases in vascular resistance could be predicted using a model involving Poiseuille's law (r^2^ = ~0.77, only one individual below 0.50). One limitation to adopting such a method is that according to Poiseuille's law, blood pressure and blood flow are positively related. This assumption ignores mechanical properties of arteries (i.e. vascular compliance and other local vasodilator and vasoconstrictor mechanisms) that modulate the relationship between pressure and flow. Of note, in the current study, during apnea blood pressure and blood flow were inversely related, likely the result of neural‐mediated increases in vascular resistance and decreases in vascular compliance. Furthermore, a combination of total MSNA and blood pressure was associated with vascular compliance during PECO. Thus, the current approach highlights the utility of examining vascular compliance, as it provides unique information regarding peripheral vascular responses to sympatho‐excitation. Nevertheless, additional studies are required to understand the unique observations that the sympathetic response to SHG has a minimal direct impact on vascular resistance or compliance.

In the present study, elevations in burst frequency and incidence during PECO did not reach statistical significance levels and total MSNA and blood pressure remained elevated whereas vascular compliance and brachial blood flow returned to baseline levels. It is important to note, although blood pressure remained elevated above baseline levels, it decreased in 5/6 participants in PECO relative to the pressor response observed during SHG. Similarly, vascular resistance also returned to baseline conditions during PECO. Thus, consistent with earlier findings, at the transition of SHG to PECO, changes in vascular mechanics and blood flow in the non‐exercising limb may be dissociated from sympathetic factors and related to changes in blood pressure (Shoemaker et al., 2000). Indeed, a linear combination of blood pressure and total MSNA served as a significant determinant of vascular compliance during PECO in the present study. Given the small sample size and that blood pressure and total MSNA are related, the precise contribution of each variable cannot be determined from the current data. Overall, the data from apnea, SHG, and PECO suggest vascular bed compliance is regulated dynamically and implicate both adrenergic and myogenic mechanisms in this phenomenon.

### 4.3 Limitations

There are limitations that should be considered when interpreting the results of the present study. Namely, this was a pilot study and the sample size consisted of a small, homogenous population of young Caucasian men and vascular regulation was only examined in the forearm vascular bed. Furthermore, because of the difficulty in assessing the independent effects of blood pressure and MSNA on vasomotor control in intact, living systems, such effects were not isolated experimentally. Therefore, the results of this study may not be generalizable to broader populations or to other vascular beds and findings pertaining to the independent effects of blood pressure and MSNA should be interpreted cautiously. In addition, the most robust changes in vascular compliance were observed during large transitory increases and not moderate steady‐state increases in sympatho‐excitation. Thus, whether differences between apnea and PECO are related to differences in sympatho‐excitation or the transitory versus steady‐state regulation of vascular compliance is unknown. Importantly, the goal of this study was not to examine differences in vascular compliance between different populations or the transitory versus steady‐state regulation, but rather to determine if the inclusion of vascular compliance adds to the understanding of neurovascular regulation. Given the large effect sizes reported (≥large effects for all indices of MSNA, brachial flow, resistance, and compliance during apnea), a large sample size was not required to examine this relationship. Thus, the present study provides the rationale for future studies to investigate differences in neural‐mediated changes in compliance across different populations as well as more mechanistic studies aimed at understanding the physiological modulators of vascular compliance.

### 4.4 Perspectives

Recent work reveals conduit artery compliance may be modulated by SNA in young adults (Holwerda et al., 2019). The present findings are the first data to our knowledge to investigate the relationship between simultaneous (transient) increases in MSNA and changes in forearm bed vascular compliance, and to examine the impact of such changes in compliance on end organ blood flow responses during different sympatho‐excitatory maneuvers. The current data collected during apnea suggest reductions in forearm vascular compliance impede flow rate, potentially through combined myogenic and α‐adrenergic mechanisms. The data collected during SHG + PECO confirm that vascular compliance and resistance operate independently to some degree, and further suggests isometric exercise and PECO uncouples the α‐adrenergic neurovascular responses. These data support model analyses that suggest SNA contributes to vascular smooth muscle cell contractility (Briant et al., 2015) as well as our hypothesis that vascular compliance contributes to changes in forearm blood flow during sympatho‐excitation in humans. In closing, we propose that efferent SNA contributes to reductions in vascular compliance, which in turn increases the opposition to pulsatile flow during apnea‐induced, but not SHG‐induced sympatho‐excitation. Furthermore, to some degree, vascular compliance and resistance operate independently from one another and that the inclusion of vascular compliance is essential in understanding both myogenic and neurogenic control of vasomotor tone and blood flow responses in the peripheral vasculature.

## CONFLICT OF INTEREST

Authors declare that there is no conflicts of interest.

## AUTHOR CONTRIBUTIONS

T. Dylan Olver, Mark B. Badrov, Matti. D Allen, Nicole S. Coverdale, and J. Kevin Shoemaker conceived the study, collected and interpreted the data. T. Dylan Olver and Mark B. Badrov analyzed the data. T. Dylan Olver drafted the manuscript and Mark B. Badrov, Matti. D Allen, Nicole S. Coverdale, and J. Kevin Shoemaker edited the manuscript.
